# Analysis of Whole-Genome for Identification of Seven *Penicillium* Species with Significant Economic Value

**DOI:** 10.3390/ijms25158172

**Published:** 2024-07-26

**Authors:** Yuanhao Huang, Lianguo Fu, Yutong Gan, Guihong Qi, Lijun Hao, Tianyi Xin, Wenjie Xu, Jingyuan Song

**Affiliations:** 1 State Key Laboratory of Bioactive Substance and Function of Natural Medicines, Institute of Medicinal Plant Development, Chinese Academy of Medical Sciences & Peking Union Medical College, Beijing 100193, China; 2 School of Life and Science, Southwest Jiaotong University, Chengdu 610031, China; 3 Key Laboratory of Chinese Medicine Resources Conservation, State Administration of Traditional Chinese Medicine of the People’s Republic of China, Engineering Research Center of Chinese Medicine Resource, Ministry of Education, Beijing 100193, China

**Keywords:** Analysis of whole-GEnome, AGE, bioinformatics analysis, Sanger sequencing, CRISPR-Cas12a, *Penicillium* species identification

## Abstract

The *Penicillium* genus exhibits a broad global distribution and holds substantial economic value in sectors including agriculture, industry, and medicine. Particularly in agriculture, *Penicillium* species significantly impact plants, causing diseases and contamination that adversely affect crop yields and quality. Timely detection of *Penicillium* species is crucial for controlling disease and preventing mycotoxins from entering the food chain. To tackle this issue, we implement a novel species identification approach called Analysis of whole GEnome (AGE). Here, we initially applied bioinformatics analysis to construct specific target sequence libraries from the whole genomes of seven *Penicillium* species with significant economic impact: *P. canescens*, *P. citrinum*, *P. oxalicum*, *P. polonicum*, *P. paneum*, *P. rubens*, and *P. roqueforti*. We successfully identified seven *Penicillium* species using the target we screened combined with Sanger sequencing and CRISPR-Cas12a technologies. Notably, based on CRISPR-Cas12a technology, AGE can achieve rapid and accurate identification of genomic DNA samples at a concentration as low as 0.01 ng/µL within 30 min. This method features high sensitivity and portability, making it suitable for on-site detection. This robust molecular approach provides precise fungal species identification with broad implications for agricultural control, industrial production, clinical diagnostics, and food safety.

## 1. Introduction

The genus *Penicillium*, comprising over 483 known species, represents a highly diverse group of fungi with wide-ranging ecological distributions across various habitats, including air, soil, indoor environments, vegetation, and food [1,2]. Among them, seven *Penicillium* species—*P. canescens*, *P. citrinum*, *P. oxalicum*, *P. polonicum*, *P. paneum*, *P. rubens*, and *P. roqueforti*—possess substantial research and application value in agriculture, industry, and medicine.

In agriculture, these species have notable impacts on plants. *P. citrinum* exhibits significant pathogenicity in post-harvest citrus fruits as a common mold, causing considerable losses [3]. *P. oxalicum* demonstrates strong antagonistic activity against plant pathogenic fungi, providing natural control of plant diseases, and is a common contaminant in spices, affecting their quality and safety [4,5,6]. *Penicillium* species are also the most common contaminants in various medicinal herbs, posing a potential threat to their safe use [6]. For instance, *P. polonicum* predominates in the contamination of medicinal seed surfaces and is a primary source of ochratoxin A contamination in licorice roots [7,8]. *P. canescens* is widely distributed in agricultural settings, particularly in harvested seeds in northwestern France, significantly hindering seed germination quality and affecting crop yields [9]. Additionally, some *Penicillium* species are associated with animal silage and spoiled food, producing harmful mycotoxins that harm livestock, human health, and food safety, indirectly impacting agricultural productivity, such as *P. paneum* and *P. roqueforti* [10,11,12,13,14,15,16,17]. Furthermore, *Penicillium* species, such as *P. citrinum*, *P. roqueforti*, and *P. rubens*, are industrially significant for producing cholesterol-lowering agents, blue cheese, penicillin, and health-beneficial natural products [18,19,20,21,22]. Clinically, however, some species, like *P. citrinum*, can act as pathogens and produce harmful carcinogenic toxins [23,24,25]. Given their significant roles in natural ecosystems and various industries, it is vital to accurately and rapidly identify these seven *Penicillium* species.

With advancements in whole genome sequencing technology, the reduction in high-throughput sequencing costs now allows for more economical direct analysis of sequencing data [26]. As a result, more genomes are being assembled and annotated for various research purposes. For example, herb genomics has been applied in traditional Chinese medicine to clarify the molecular mechanisms involved in the prevention and treatment of human diseases [27]. A significant number of fungal genomes are now publicly accessible, with the number of published fungal genomes rising to 18,023 as of 12 June 2024, according to the National Center for Biotechnology Information (NCBI) (https://www.ncbi.nlm.nih.gov, accessed on 12 June 2024). Fungal genomes are a crucial resource, not only for the synthesis and pathway elucidation of species-specific metabolites but also for interpreting phylogenetic relationships within fungal lineages [20,28]. Automated phylogenetic pipelines based on core genes have been widely adopted in the prokaryotic kingdom [28], such as the Genome Taxonomy Database (GTDB) [29], AutoMLST [30], or UBCG [31].

**Figure 1:**
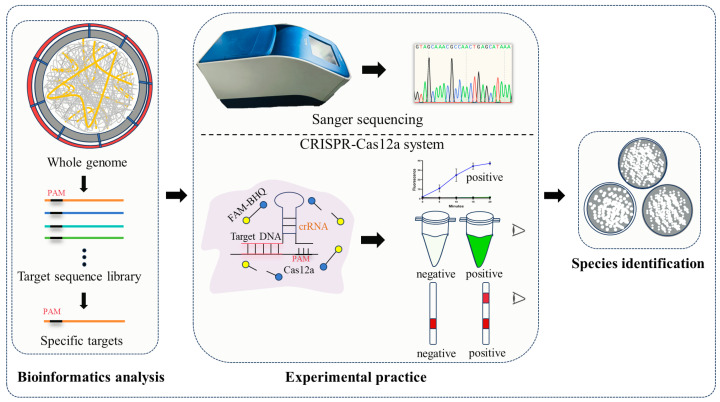
The process of AGE. Firstly, bioinformatic analysis is used to screen 25 bp sequences containing protospacer adjacent motifs (PAM) to construct a target sequence library from whole genome, followed by further selection of specific targets. Subsequently, Sanger sequencing and CRISPR-Cas12a experimental technologies are employed to validate these specific targets, facilitating the identification of *Penicillium* species. Notably, AGE-integrated CRISPR-Cas12a technology allows for the visualization of identification results.

In this study, we employed a novel approach called Analysis of whole GEnome (AGE) to identify *Penicillium* species. The fundamental principle of AGE is that the genome of each species is unique, enabling the identification of interspecies genomic differences across the whole genome [32]. The AGE process initially involves bioinformatics analysis utilized to select specific target sequences that are conserved within the target species and specific interspecifically from the whole genome, followed by experimental validation of the target using technologies (Figure 1), which has been preliminarily utilized for species identification of *Crocus sativus* [33], *Cervus nippon*, and *Cervus elaphus* [34], 13 fungal species [35], and closely related *Aspergillus* species [36]. In our study, we first conducted an identification study of seven *Penicillium* species and constructed their target libraries from whole genomes. This was motivated by their significant economic value, the abundance of genomic data, and their availability in culture collections. We used Sanger sequencing and CRISPR-Cas12a to demonstrate that the targets we screened could be successfully utilized for species identification. Notably, based on CRISPR-Cas12a technology, detection results could be visualized within 30 min. This highlights its potential for on-site detection, providing robust support to address the need for rapid and accurate species identification in agricultural control, industrial production, clinical diagnostics, food and drug safety, and fungal taxonomy.

## 2. Results

### 2.1. Bioinformatics Analysis Screening of Species-Specific Targets for Seven Penicillium Species Identification

**Figure 2:**
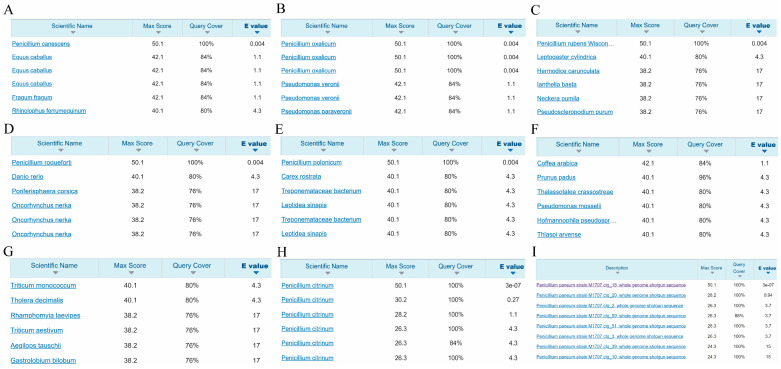
Target BLAST results of seven *Penicillium* species. (**A**) The BLAST result of Pcan_target in the NCBI Nucleotide Blast database. (**B**) The BLAST result of Pox_target in the NCBI Nucleotide Blast database. (**C**) The BLAST result of Prub_target in the NCBI Nucleotide Blast database. (**D**) The BLAST result of Proq_target in the NCBI Nucleotide Blast database. (**E**) The BLAST result of Ppol_target in the NCBI Nucleotide Blast database. (**F**) The BLAST result of Pcit_target in the NCBI Nucleotide Blast database. (**G**) The BLAST result of Ppan_target in the NCBI Nucleotide Blast database. (**H**) The BLAST result of Pcit_target in the genome of *P. citrinum*. (**I**) The BLAST result of Ppan_target in the genome of *P. paneum*.

In order to screen suitable species-specific targets for identifying seven *Penicillium* species, we utilized bioinformatics analysis to extract 25 bp sequences containing protospacer adjacent motifs (PAM) from the whole genomes of the seven *Penicillium* species. At this moment, seven species have 25 bp sequences containing PAM, ranging from 688,324 to 1,769,667 (Supplementary Table S1). Subsequently, we conducted a comparative analysis of these 25 bp sequences and screened species-specific sequences to construct target libraries for each species. Each target in the library exhibited more than three mismatches and all indels within 21 bp, excluding the PAM sequence, to ensure specificity across the seven species. The range of high-frequency targets (≥50%) for seven species is 249,488 to 772,043 (Supplementary Table S1), and the high-frequency targets of all seven species are distributed on all chromosomes and over 95% of the scaffolds. We randomly selected one high-frequency target for NCBI BLAST from each library, which were named Pcan_target, Pox_target, Pcit_target, Ppan_target, Proq_target, Prub_target, and Ppol_target (Table 1). BLAST results revealed that five targets exhibited high specificity and were perfectly aligned with the target species (Figure 2A–E). Notably, the Pcit_target and Ppan_target did not individually align with the target species *P. citrinum* and *P. paneum* in the NCBI Nucleotide Blast database (Figure 2F,G), but they successfully aligned with the genome of the target species (Figure 2H,I). Furthermore, we also successfully aligned Pcit_target and Ppan_target in all genomes of their respective target species (Supplementary Table S2).

### 2.2. Sequencing Successfully Identified Target Species

**Figure 3:**
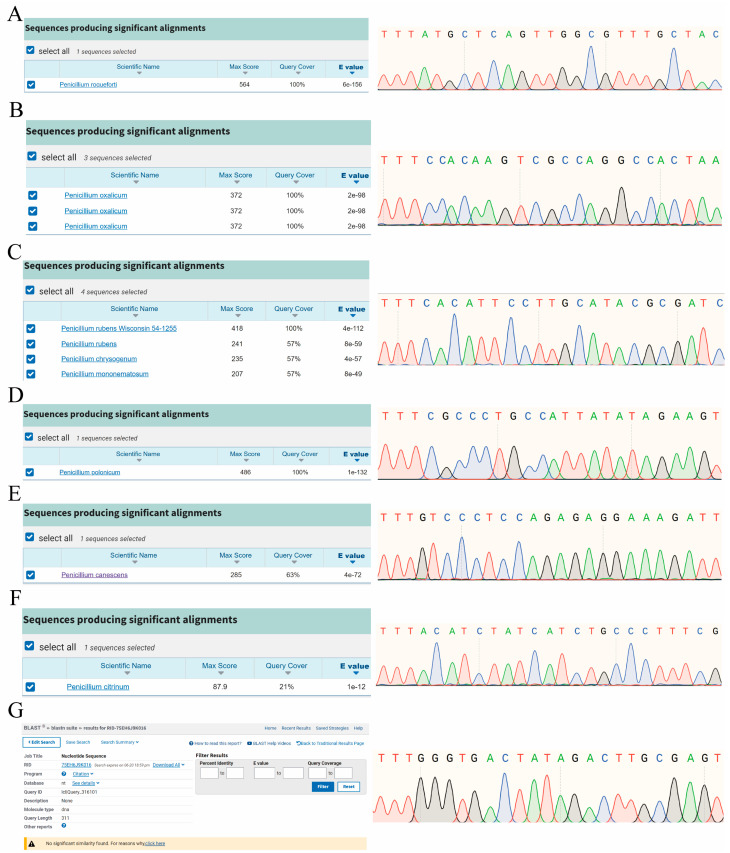
Sanger sequencing and amplicon sequences of targets NCBI BLAST results of seven *Penicillium* species. (**A**–**G**), respectively, represent the BLAST results of the sequencing and amplicon sequences of targets for *P. roqueforti*, *P. oxalicum*, *P. rubens*, *P. polonicum*, *P. canescens*, *P. citrinum*, and *P. paneum*.

In this study, we used Sanger sequencing to validate seven targets. Primers were designed to amplify the genomic DNA of seven *Penicillium* species (Supplementary Table S3). Sanger sequencing results demonstrated that the targets were fully amplified and successfully aligned in the target species, with high sequencing quality (Figure 3). Upon aligning the amplified targets with the NCBI database, we found that amplified sequences aligned with the target species and exhibited high specificity (Figure 3A–E), showing more than three nucleotide differences with other species.

Notably, the BLAST results of the amplification sequence containing Pcit_target covered only 21% of the sequence length (Figure 3F). The aligned amplification sequence corresponds to a partial mRNA sequence (Supplementary Figure S1) but does not contain Pcit_target. (Supplementary Figure S1). However, its amplification sequence is present in the target species genome (Supplementary Figure S1). Similarly, the amplicon containing the Ppan_target did not align with any species in the NCBI Nucleotide Blast database (Figure 3G), but it is present in the target species genome (Supplementary Figure S1).

Furthermore, seven targets were used to query the NCBI database, revealing intriguing findings. Pcan_target, Pox_target, Pcit_target, and Ppol_target were located in the annotated region of their genome, while Ppan_target, Proq_target, and Prub_target did not have any annotation information (Supplementary Table S4). This finding underscores the applicability of the target in both unannotated and annotated regions in species identification.

### 2.3. CRISPR-Cas12a Technology Rapidly Detected Target Species

**Figure 4:**
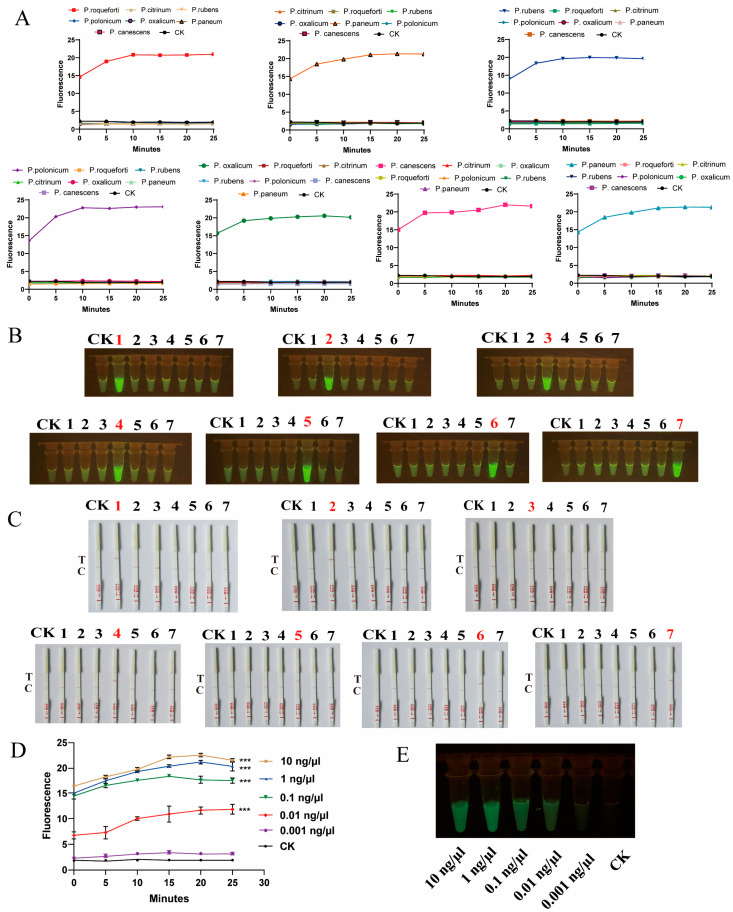
AGE achieved the precise species identification of seven *Penicillium* species using the CRISPR-Cas12a technology. (**A**–**C**): AGE achieved the precise identification of each target species, respectively, using fluorescence, visible fluorescence, and lateral flow strip, without cross-reaction with other *Penicillium* species. (**D**,**E**): The sensitivity detection, respectively, by fluorescence and visual fluorescence. CK: nuclease-free water. Error bars represented means ± standard deviation (SD) from three independent replicates. ***, *p* < 0.001. Each group contains all the reagents of the CRISPR system with different DNA substrates. 1: *P. roqueforti*; 2: *P. citrinum*; 3: *P. rubens*; 4: *P. polonicum*; 5: *P. oxalicum*; 6: *P. canescens*; and 7: *P. paneum*. T: text line; C: control line. The presence of T indicates a positive result, whereas the presence of only C indicates a negative result.

Following the successful validation of targets for the identification of seven *Penicillium* species via Sanger sequencing, we utilized CRISPR-Cas12a technology integrated with room-temperature amplification to achieve rapid identification of seven *Penicillium* species, presenting results in three formats: fluorescence values, visible fluorescence, and lateral flow strips (Figure 4A–C). The results consistently indicated accurate identification of the *Penicillium* target species without cross-reactivity with other species, demonstrating the high specificity of the targets. Notably, CRISPR-Cas12a technology enables rapid and visual detection. The visible fluorescence results can be observed with the naked eye using a simple blue light instrument (Figure 4B). Concurrently, the lateral flow strip results offer greater convenience, allowing for result determination based on the test line (Figure 4C).

**Figure 5:**
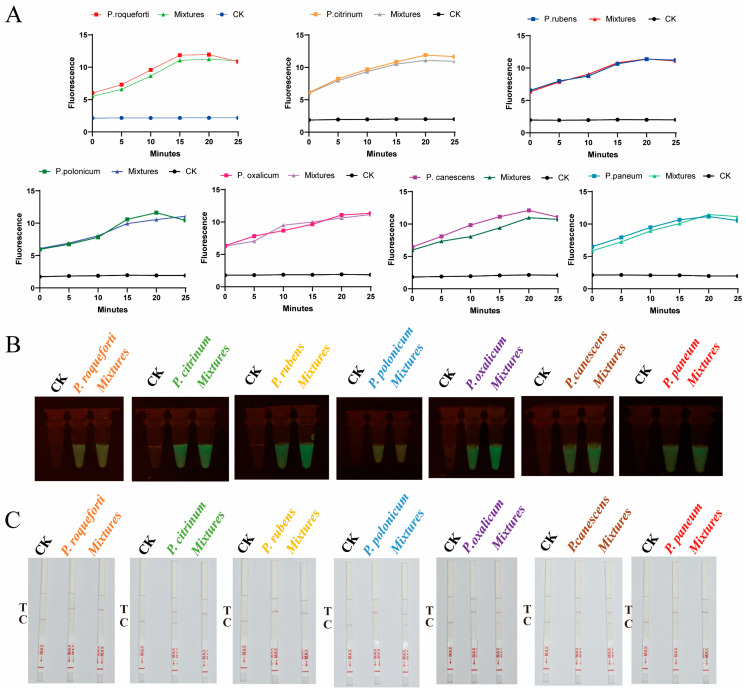
AGE precisely identified seven *Penicillium* target species in mixture samples. (**A**–**C**): AGE achieved target species identification with fluorescence, visible fluorescence, and lateral flow strip. Mixtures: one target species and other six species. CK: mixtures without target species. T: text line; C: control line. The presence of T indicates a positive result, whereas the presence of only C indicates a negative result.

To evaluate the detection sensitivity of CRISPR-Cas12a, we prepared genomic DNA of *P. oxalicum* at five different DNA concentrations, ranging from 0.001 to 10 ng/μL. The t-test showed that there was a significant difference between samples with DNA concentrations equal or greater than 0.01 ng/μL and the control group (CK) during the 25 min, but no significant difference was found between DNA concentrations 0.001 ng/μL and CK. Moreover, CRISPR-Cas12a demonstrated excellent stability, with the fluorescence values remaining relatively constant. Therefore, the limit of detection (LOD) of CRISPR-Cas12a was considered as 0.01 ng/μL (Figure 4D). Moreover, when the concentration was lower than 0.01 ng/μL, the visible fluorescence detection results appeared dimmer (Figure 4E). In conclusion, AGE can effectively detect DNA concentrations as low as 0.01 ng/μL, visually displaying results of visible fluorescence and lateral flow strip within 30 min. Additionally, in practical settings, most samples are mixed, containing more than one fungal species. Therefore, to assess the specificity of AGE for detecting target species in mixed samples, we conducted mixed detection of seven *Penicillium* species (Figure 5). We found that AGE successfully detected the target in artificially mixed sample DNA, unaffected by the presence of diverse DNA samples.

## 3. Discussion

### 3.1. AGE Enables Construct-Specific Target Libraries for Seven Penicillium Species Identification

*Penicillium* species are among the most prevalent fungi worldwide [37]. They interact significantly with plants and can occasionally act as pathogens in human and animal mycoses, with substantial economic implications [38,39]. Therefore, species-level identification has been a cornerstone of biology [40]. In this study, we selected seven *Penicillium* species with important economic value and utilized AGE for their identification. Reliable genomes of these species were downloaded for bioinformatics analysis to construct target libraries. The high-frequency specific target number ranges from 249,488 to 772,043. These abundant targets provide support for species identification. Randomly selected one high-frequency target from per library matched well with the target species and exhibited more than three nucleotide differences from other species according to NCBI BLAST, demonstrating broad specificity. We successfully identified them via Sanger sequencing and CRISPR-Cas12a.

Interestingly, of the seven targets involved in this study, Ppan_target, Proq_target, and Prub_target are situated within the unannotated segment of their genome. These totally new sequences were called “dark matter” in the genome. Additionally, there are four targets with annotated information that were not utilized for the identification of species. Pcan_target is located within the coding sequence of cytochrome P450. Cytochrome P450 is a large, evolutionarily conserved superfamily of membrane-bound heme proteins found in animals, plants, and microorganisms [41,42]. These proteins are primarily studied for their role in catalyzing the metabolism of various substances [43]. Pox_target and Ppol_target are situated in a hypothetical gene. Hypothetical proteins, due to the absence of known homologous genes, remain enigmatic regarding their potential to encode actual proteins [44]. Pcit_target is found within the gene region of chromatin modification-related protein eaf3. Protein eaf3 is a crucial regulator of acetylation and is primarily studied for its role in binding key active substances [45,46]. Our results indicated that targets, whether located in annotated or unannotated regions of the genome, have significant potential for species identification. AGE enables the accurate screening of species-specific targets from the whole genome by combining bioinformatics analysis, providing a scientific basis for distinguishing different fungi.

### 3.2. AGE-Integrated CRISPR-Cas12a Technology for Rapid and Accurate Species On-Site Detection

Nucleic acid-based identification techniques typically involve three main steps: nucleic acid extraction, amplification, and product detection. Among these, detection is particularly crucial as it directly reveals the results of the assay [47]. Recent advancements have successfully employed the CRISPR-Cas12a system for the precise detection of bacteria [48] and viruses [49]. Additionally, over the past decades, significant research has focused on isothermal amplification methods, including Recombinase Polymerase Amplification (RPA) [50], loop-mediated isothermal amplification (LAMP) [51], and Enzymatic Recombinase Amplification (ERA) [52].

In current and future studies of fungi, rapid and accurate detection technologies are indispensable. Our study demonstrates the potential of AGE for on-site fungal detection. AGE incorporates advanced technologies, including CRISPR-Cas12a and ERA, to support a range of detection formats, such as fluorescence intensity, visible fluorescence, and lateral flow strip assays. The latter two formats, in particular, offer the advantages of portability and suitability for on-site applications without requiring costly and precise instruments. Remarkably, accurate detection of seven *Penicillium* species can be achieved with DNA concentrations as low as 0.01 ng/μL within 30 min, even in mixed samples, meeting the urgent needs of clinical diagnostics, customs inspections, and market surveillance for rapid fungal identification. For effective on-site detection, rapid nucleic acid extraction technologies are also critical. For example, disposable polymeric microneedle (MN) patches can efficiently extract DNA from various plant leaf samples in under a minute [53], providing DNA suitable for nucleic acid amplification analysis. It is imperative to thoroughly study and optimize all stages of nucleic acid extraction, amplification, and product detection. In the future, AGE aims to integrate a suite of innovative technologies to enhance the on-site detection of fungal species.

### 3.3. Challenge and Development of AGE

Despite demonstrating significant potential in accurate species identification, the application of AGE in this study still faces limitations and necessitates further efforts. Firstly, according to the NCBI database, there are 531 *Penicillium* genomes available, encompassing 108 species as of 12 June 2024. In this study, we selected seven economically important *Penicillium* species and constructed target libraries for them. Close phylogenetic relationships within the genus can potentially affect target specificity. To ensure that AGE can be accurately applied to the entire *Penicillium* genus, it is essential to include more *Penicillium* genomes in bioinformatic analyses to establish comprehensive target libraries for the genus in the future. Moreover, DNA barcoding has become the most widely used molecular identification technique [54,55,56,57], with the internal transcribed spacer region (ITS) being selected as the universal barcoding for fungi [58]. However, the ITS sequence is not equally variable in all groups of fungi [59], particularly within the genus *Penicillium* [60,61]. Therefore, precise identification of *Penicillium* species is crucial. AGE can screen targets from the whole genome, successfully discriminating among closely related species where ITS-based identification faces challenges [36]. By constructing target libraries for the entire *Penicillium* genus across the whole genome rather than being confined to specific regions, AGE will provide abundant target information to address the identification challenge of *Penicillium* species.

Secondly, although we confirmed that the targets we selected can accurately identify seven *Penicillium* species and exhibit broad specificity through NCBI BLAST, we lack additional *Penicillium* species samples for specific validation. Therefore, it is necessary to collect more other *Penicillium* species for experimental practice to ensure that the targets are highly specific.

Lastly, it is vital for the accuracy of AGE to select reliable genomes for bioinformatic analysis. There are instances of misclassified genomes in the NCBI database, such as *P. solitum* (RS1), which should be classified as *P. polonicum* (RS1) [62]. In this study, we also confirmed that Ppol_target successfully aligns with *P. solitum* (RS1) (Supplementary Figure S1). To avoid the impact of unreliable genomes on the results, this study selected reliable species genomes of seven *Penicillium* species based on one of three criteria: the authority of the submitting strain’s genome unit, whether it is a standard strain, and the quality and number of articles published about the strain. Reliable genomes are the cornerstone for the precise species identification of targets using AGE. Therefore, researchers must ensure the accuracy of species genome submissions.

## 4. Materials and Methods

### 4.1. Strain Preparation and DNA Extraction

In this study, seven *Penicillium* species standard strains were selected for AGE identification research: *P. canescens*, *P. citrinum*, *P.oxalicum*, *P. polonicum*, *P. paneum*, *P. roqueforti*, and *P. rubens*, which were acquired from the Shanghai Microorganism Culture Collection Center (SHMCC, Shanghai, China) (Supplementary Table S5). The fungal samples were cultured on PDA medium, and approximately 50 mg of fresh mycelium was collected, immersed in liquid nitrogen, and ground into a fine powder. Genomic DNA was then extracted using the commercial genomic DNA extraction kit (DP305, Tiangen Biotech (Beijing) Co., Ltd., Beijing, China), according to the manufacturer’s protocols. The integrity of the extracted genomic DNA was assessed by 1% agarose gel electrophoresis in 1× TAE buffer at 140 V for 40 min (Bio-Rad Laboratories Inc., Hercules, CA, USA), followed by purity and concentration evaluation using the Nanodrop 2000 spectrophotometer (Thermo Fisher Scientific, Waltham, MA, USA). The extracted DNA was stored at −80 °C until further use.

### 4.2. Screening Species-Specific Targets with Bioinformatics Analysis

The genomes of all analyzed species were downloaded from the NCBI database (https://www.ncbi.nlm.nih.gov, accessed on 12 June 2024), with reliable genome versions listed in Supplementary Table S6. The genomes were fragmented into 25 bp segments using Jellyfish (v1.1.12). The 25 bp containing PAM sequences (TTTV at the start or VAAA at the end) were extracted and compared to the genomes themselves using Bowtie (v1.1.0) with zero mismatches in setting to determine their locations within their respective genomes. Subsequently, all 21 bp sequences, excluding the PAM, were aligned to those of other species to create a target library using Bowtie (v1.1.0) with three mismatches in setting. All selected sequences were considered potential targets. Additionally, we analyzed high-frequency targets from the potential targets to ensure their conservativeness in the species’ genomes. We selected high-frequency conserved targets for alignment in NCBI, and if it aligned with non-target species, we would reselect new targets for analysis to ensure the targets’ specificity. Among these, a specific target was chosen for the design of crRNAs as guided by referenced literature [63,64] (Supplementary Table S3).

### 4.3. Identification of Species-Specific Targets

#### 4.3.1. Recognition of Specific Targets via Sanger Sequencing

We designed specific primers for each species based on the target region flanked by 300 bp upstream and downstream using Primer Premier6.0 software. These primers were synthesized by GenScript Co., Ltd., Nanjing, China, and used to amplify the target region. PCR amplification was performed in a 25 μL reaction volume containing 2× Taq MasterMix (12.5 μL, Aidlab Biotechnologies Co., Ltd., Beijing, China), 1 μL F/R primer (10 μmol/L), 2 μL DNA template (10 ng/µL), and 8.5 µL nuclease-free water. Samples were amplified in an Applied Biosystems Veriti™ Thermal Cycler (Thermo Fisher Scientific, Waltham, MA, USA), and the PCR program was set as follows: initial denaturation at 94 °C for 5 min, followed by 30 cycles of 94 °C for 1 min, 55 °C for 30 s, 72 °C for 1.5 min, and a final extension at 72 °C for 10 min. The amplified DNA was purified according to the instructions of the QIAquick PCR Purification Kit (Qiagen Co., Ltd., Hilden, Germany), and the purified PCR products were sequenced by Sanger bidirectional sequencing on an ABI 3730 XL analyzer. Contig assembly and consensus sequence generation were carried out using CodonCode Aligner software (version 10.0.3), and low-quality sequence data and primer sequences were removed. Sequence alignments were performed using the BLAST program on the NCBI website.

#### 4.3.2. CRISPR-Cas12a-Based Detection of Specific Targets

This study employed ERA, an isothermal PCR alternative, enabling amplification at room temperature. The rapid identification system involves two steps: amplification and detection step. The total volume of the amplification reaction system was 50 µL, comprising 43 µL ERA amplification mix (Suzhou Xinda Gene Technology Co., Ltd., Suzhou, China), 2.5 µL F/R primer, and 2 µL DNA (10 ng/µL). The detection system also has a total volume of 50 µL, including 10 µL 10× NEBuffer 2.1 (New England Biolabs, (Beijing) Ltd., Beijing, China), 2 µL Cas12a (20 nM, New England Biolabs, Ipswich, MA, USA), 3.3 µL crRNA (300 nM, Nanjing KingsRay Biotechnology Co., Ltd., Nanjing, China), 4 µL ssDNA (/5′6-FAM/CCCCCCCCCC/3′BHQ-1, 400 nM, Suzhou Jinweizhi Biotechnology Co., Ltd., Suzhou, China) or 80 nM FAM-Biotin fluorescent signal molecule (/5′6-FAM/TTATTATT/3′Bio, Nanjing KingsRay Biotechnology Co., Ltd., Nanjing, China), and 30.7 µL nuclease-free water. The ERA amplification reagent, DNA, and primers are incubated at 37 °C for 20 min. Following this, NEBuffer 2.1, Cas12a, crRNA, and nuclease-free water are added, and the mixture is further incubated at 37 °C for 10 min. Finally, ssDNA is added for fluorescence value and visual fluorescence detection. The fluorescence intensity is measured using a microplate reader (Thermo Fisher Scientific) at λ_ex 483 nm/λ_em 535 nm, and the visual fluorescence is directly observed under a blue light illuminator. When we integrated ambient temperature amplification with the CRISPR-Cas12a system and lateral flow strip assay (TS104, Suzhou GenDx Biotech Co., Ltd., Suzhou, China) for the specific identification of targets, we substituted ssDNA with an 80 nM FAM-Biotin fluorescent signal molecule (/5′6-FAM/TTATTATT/3′Bio). Subsequently, the lateral flow strip was inserted into a centrifuge tube containing the reactants to observe the results. The amplified products were recognized by the biotin ligand and the anti-FAM monoclonal antibody present on the test line, where gold nanoparticles have been immobilized. The sticks exhibited two lines: a test line and a control line, with the appearance of both lines indicating a positive result [65].

### 4.4. Detection of Penicillium to Assess Specificity and Sensitivity

To validate the specificity of the established AGE method, we analyzed DNA samples from seven *Penicillium* species, using 2 μL of each sample as a template. Target identification was achieved through the CRISPR-Cas12a system. To determine the sensitivity of the AGE method in detecting *Penicillium* species, we selected *P. oxalicum* as a case study. The assay involved testing genomic DNA at six concentration levels: 10 ng/μL, 1 ng/μL, 0.1 ng/μL, 0.01 ng/μL, and 0.001 ng/μL, using 2 μL from each concentration as the template. The results were visualized by measuring fluorescence intensity and visible fluorescence through the CRISPR-Cas12a system.

### 4.5. Mixture Sample Detection for Penicillium Species

To facilitate field detection, we conducted comparative analyses by testing mixed samples containing the target species, mixed samples without the target species, and samples containing only the target species, aiming to simulate whether species diversity in real samples would interfere with the detection of target species. The total volume of the mixed sample is 21 µL, which includes 3 µL of DNA (0.1 ng/µL) from each species, with nuclease-free water added to CK to reach the final volume. A 2 µL mixed sample is used for CRISPR-Cas12a detection, following the procedure outlined in Section 4.3.2.

## 5. Conclusions

In this study, we first constructed target libraries of seven *Penicillium* species from whole genomes and achieved accurate identification of them. Furthermore, we demonstrated the significant identification potential of the target in both annotated and non-annotated regions. Notably, based on CRISPR-Cas12a and room-temperature amplification, AGE can achieve visual results within 30 min, enabling on-site detection. This will provide robust support for subsequent on-site fungal detection.

## Figures and Tables

**Table 1:** Targets for identification of seven *Penicillium* species.

Species	No.	Specific Target (5′→3′)
*P. canescens*	Pcan_target	TTTGTCCCTCCAGAGAGGAAAGATT
*P. oxalicum*	Pox_target	TTTCCACAAGTCGCCAGGCCACTAA
*P. citrinum*	Pcit_target	TTTACATCTATCATCTGCCCTTTCG
*P. paneum*	Ppan_target	TTTGGGTGACTATAGACTTGCGAGT
*P. roqueforti*	Proq_target	TTTATGCTCAGTTGGCGTTTGCTAC
*P. rubens*	Prub_target	TTTCACATTCCTTGCATACGCGATC
*P. polonicum*	Ppol_target	TTTCGCCCTGCCATTATATAGAAGT

## Data Availability

Data are available within the article or its Supplementary Materials.

## Supplementary Materials

The following supporting information can be downloaded at https://www.mdpi.com/article/10.3390/ijms25158172/s1.
