# Supplementary material for: Analysis of Whole-Genome for Identification of Seven Penicillium Species with Significant Economic Value
Source: Int J Mol Sci. 2024 Jul 26;25(15):8172. doi: 10.3390/ijms25158172 (PMC11312406; doi:10.3390/ijms25158172)
Supplement: Supplementary file 1 [file ijms-25-08172-s001.zip › ijms-3093882-supplementary.pdf]

**Supplementary legends**

**Supplementary Table S1 to S6**

Supplementary Table S1 The bioinformatics results of target number for seven *Penicillium* species.

Supplementary Table S2 The BLAST results of the Pcit_target and Ppan_target within the all genomes of their target species.

Supplementary Table S3 The primers and crRNAs of seven *Penicillium* species.

Supplementary Table S4 The information of the targets within the genome.

Supplementary Table S5 The strain purchase ID of seven *Penicillium* species.

Supplementary Table S6 The reliable genome accession number of seven *Penicillium* species in NCBI GenBank.

**Supplementary Figure S1**

The BLAST result in NCBI database. (A) The amplification sequence containing the Pcit_target aligned to a partial mRNA sequence. (B) The BLAST result of the amplification sequence on NCBI database do not contain Pcit_target. (C) The BLAST analysis of the 47 bp amplification sequence from *P. citrinum* in the NCBI database showed alignment with the Pcit_target. (D) The amplification sequence containing the Pcit_target is present in the genome of the *P. citrinum.* (E) The BLAST result of amplicon containing the Ppan_target in the genome of *P. paneum*. (F) The BLAST result of Ppol_target in the genome of *P. solitum* (RS1).

**Supplementary Information**

**Table S1.** The bioinformatics results of target number for seven *Penicillium* species.

| **Species** | **25-bp sequences containing PAM** | **High-frequency shared target number (f ≥0.5)** | **Proportion of high-frequency shared target** | **High-frequency specific target number (f ≥0.5)** | **Proportion of high-frequency specific target** |
|---|---|---|---|---|---|
| *Penicillium canescens* | 1769667 | 915946 | 52% | 730889 | 41% |
| *Penicillium oxalicum* | 758534 | 758534 | 100% | 646645 | 85% |
| *Penicillium citrinum* | 1143284 | 908976 | 80% | 772043 | 68% |
| *Penicillium paneum* | 688324 | 688324 | 100% | 249488 | 36% |
| *Penicillium roqueforti* | 1102165 | 702132 | 64% | 256542 | 23% |
| *Penicillium rubens* | 908144 | 908144 | 100% | 490679 | 54% |
| *Penicillium polonicum* | 1380642 | 749816 | 54% | 410040 | 30% |

f indicates that frequency of targets at 50% or above. Targets number indicates unique targets number.

**Table S2.** The BLAST results of the Pcit_target and Ppan_target within the genomes of their target species.

| Species | GenBank accession number | The BLAST results |
|---|---|---|
| *Penicillium citrinum* | GCA_020284165.1 | √ |
| | GCA_036320845.1 | √ |
| | GCA_023624595.1 | √ |
| | GCA_001950535.1 | √ |
| | GCA_025768555.1 | √ |
| | GCA_019191195.1 | √ |
| | GCA_025768405.1 | √ |
| | GCA_025768355.1 | √ |
| | GCA_025782865.1 | √ |
| | GCA_025768795.1 | √ |
| | GCA_025768705.1 | √ |
| | GCA_025768525.1 | √ |
| | GCA_025768585.1 | √ |
| | GCA_025768325.1 | √ |
| | GCA_037044215.1 | √ |
| | GCA_025768385.1 | √ |
| | GCA_025768765.1 | √ |
| | GCA_025768735.1 | √ |
| | GCA_025768665.1 | √ |
| | GCA_025768645.1 | √ |
| | GCA_023624675.1 | √ |
| | GCA_025768615.1 | √ |
| | GCA_025768445.1 | √ |
| | GCA_027569755.1 | √ |
| | GCA_025531905.1 | √ |
| | GCA_023624655.1 | √ |
| *Penicillium paneum* | GCA_001399475.1 | √ |
| | GCA_023624635.1 | √ |
| | GCA_023624615.1 | √ |
| | GCA_023624515.1 | √ |
| | GCA_023624575.1 | √ |
| | GCA_023624495.1 | √ |
| | GCA_023624535.1 | √ |
| | GCA_023624555.1 | √ |
| | GCA_000577715.1 | √ |

√ indicates that the target successfully aligns with the genome.

**Table S3.** The primers and crRNAs of seven *Penicillium* species.

| Species | Primers | crRNA (5′→3′) |
|---|---|---|
| *Penicillium canescens* | F: TCTCATTCTCAGTCATTCATTCCCG<br>R: ACACTTCCAATGTACTTAGTGTCGC | UAAUUUCUACUAAGUGUAGAU<br>UCCCUCCAGAGAGGAAAGAUU |
| *Penicillium oxalicum* | F: TTGCTTCCACTTGACAAGATACTGC<br>R:ACAGAGGATATGCAGGACACTTCAG | UAAUUUCUACUAAGUGUAGAU<br>CACAAGUCGCCAGGCCACUAA |
| *Penicillium citrinum* | F:AGACTTGTTGCAACGTGAATGCAGC<br>R: AAGAACCCTCTCGAGTTTCTCGTCC | UAAUUUCUACUAAGUGUAGAU<br>CAUCUAUCAUCUGCCCUUUCG |
| *Penicillium paneum* | F: AGTCGGAAGCAATCACGTCGACTTG<br>R: TGGTTGAGAGTCCCACAGACAATCC | UAAUUUCUACUAAGUGUAGAU<br>GCCCUGCCAUUAUAUAGAAGU |
| *Penicillium roqueforti* | F: TGCATTAGGAAGTAGCAGGAAGTAG<br>R: TTTCGAGGGTGAAAGGTAACGACAG | UAAUUUCUACUAAGUGUAGAU<br>UGCUCAGUUGGCGUUUGCUAC |
| *Penicillium rubens* | F: TTGATGTATCAATCGGTCCAGATCC<br>R: AGAGTACTTCATAGCCTCTTCTTGG | UAAUUUCUACUAAGUGUAGAU<br>ACAUUCCUUGCAUACGCGAUC |
| *Penicillium polonicum* | F: TCTTCTAGTGAGTTTGTTGCGTCTC<br>R: ATGCGTGACTTTAGTTTCATCACGC | UAAUUUCUACUAAGUGUAGAU<br>GCCCUGCCAUUAUAUAGAAGU |

**Table S4.** The information of the targets within the genome.

| Species | Target Location | Annotation |
|---|---|---|
| *Penicillium canescens* | unknown | cytochrome P450 |
| *Penicillium citrinum* | unknown | chromatin modification-related protein eaf3 |
| *Penicillium oxalicum* | chromosome I | hypothetical protein |
| *Penicillium polonicum* | unknown | hypothetical protein |
| *Penicillium paneum* | unknown | unannotated |
| *Penicillium roqueforti* | unknown | unannotated |
| *Penicillium rubens* | unknown | unannotated |

**Table S5.** The strain purchase ID of seven *Penicillium* species.

| Species | Strain Purchase ID |
|---|---|
| *Penicillium canescens* | SHBCC D23021 |
| *Penicillium citrinum* | SHBCC D11600 |
| *Penicillium oxalicum* | SHBCC D19274 |
| *Penicillium polonicum* | SHBCC D22498 |
| *Penicillium paneum* | SHBCC D28331 |
| *Penicillium roqueforti* | SHBCC D11584 |
| *Penicillium rubens* | SHBCC D22391 |

**Table S6.** The reliable genome accession number of seven *Penicillium* species in NCBI GenBank.

| Species | GenBank Accession Number |
|---|---|
| *Penicillium canescens* | GCA_028828765.1 |
| *Penicillium canescens* | GCA_028828735.1 |
| *Penicillium canescens* | GCA_028828745.1 |
| *Penicillium canescens* | GCA_028828795.1 |
| *Penicillium canescens* | GCA_028828825.1 |
| *Penicillium canescens* | GCA_028829005.1 |
| *Penicillium canescens* | GCA_028829745.1 |
| *Penicillium oxalicum* | GCA_021133555.1 |
| *Penicillium citrinum* | GCA_028827155.1 |
| *Penicillium citrinum* | GCA_020284165.1 |
| *Penicillium citrinum* | GCA_027569755.1 |
| *Penicillium paneum* | GCA_000577715.1 |
| *Penicillium roqueforti* | GCA_023065415.1 |
| *Penicillium roqueforti* | GCA_023065495.1 |
| *Penicillium roqueforti* | GCA_023065595.1 |
| *Penicillium roqueforti* | GCA_023137995.1 |
| *Penicillium roqueforti* | GCA_023138355.1 |
| *Penicillium roqueforti* | GCA_023138445.1 |
| *Penicillium roqueforti* | GCA_023138615.1 |
| *Penicillium roqueforti* | GCA_023138675.1 |
| *Penicillium roqueforti* | GCA_023141305.1 |
| *Penicillium roqueforti* | GCA_023141315.1 |
| *Penicillium rubens* | GCA_028828025.1 |
| *Penicillium rubens* | GCA_028828505.1 |
| *Penicillium polonicum* | GCA_025589915.1 |
| *Penicillium polonicum* | GCA_025590805.1 |
| *Penicillium polonicum* | GCA_027569845.1 |

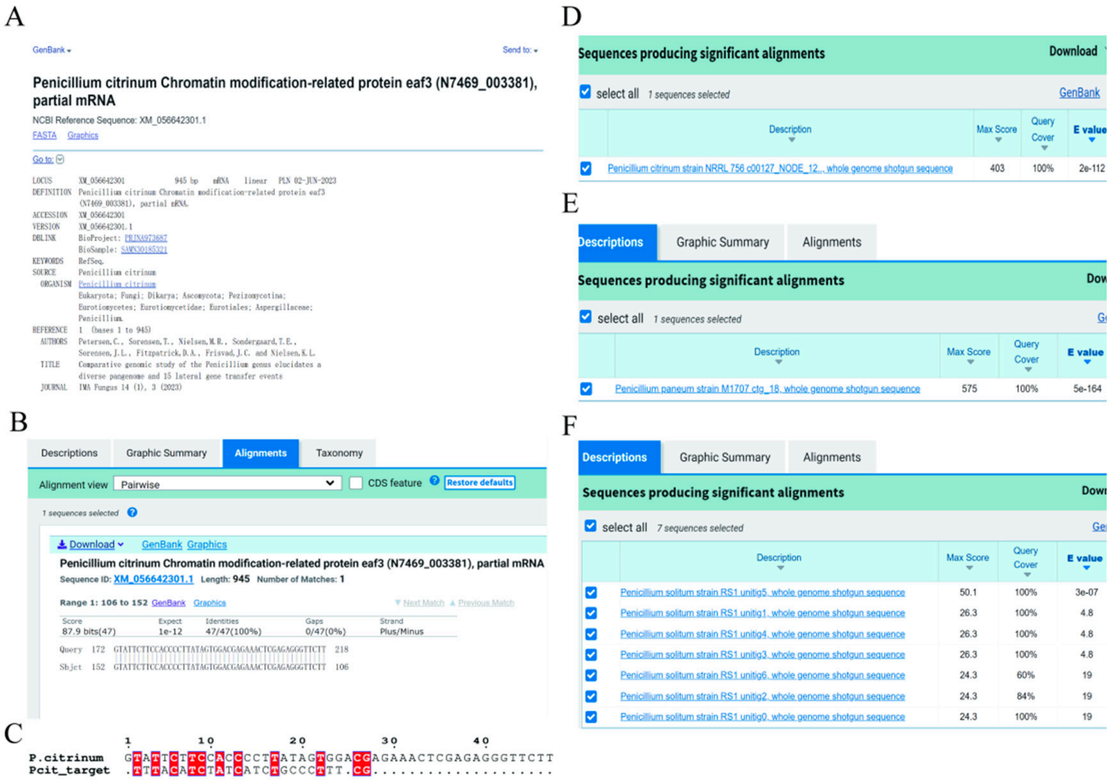


**Figure S1.** The BLAST result in NCBI database. (A) The amplification sequence containing the Pcit_target aligned to a partial mRNA sequence. (B) The BLAST result of the amplification sequence on NCBI database do not contain Pcit_target. (C) The BLAST analysis of the 47 bp amplification sequence from *P. citrinum* in the NCBI database showed alignment with the Pcit_target. (D) The amplification sequence containing the Pcit_target is present in the genome of the *P. citrinum*. (E) The BLAST result of amplicon containing the Ppan_target in the genome of *P. paneum*. (F) The BLAST result of Ppol_target in the genome of *P. solitum* (RS1).
